# The Accuracy of Cerenkov Photons Simulation in Geant4/Gate Depends on the Parameterization of Primary Electron Propagation

**DOI:** 10.3389/fphy.2022.891602

**Published:** 2022-04-28

**Authors:** Carlotta Trigila, Gerard Ariño-Estrada, Sun Il Kwon, Emilie Roncali

**Affiliations:** 1 Department of Biomedical Engineering, University of California Davis, Davis, CA, United States; 2 Department of Radiology, University of California Davis, Davis, CA, United States

**Keywords:** Cerenkov photons, timing resolution, positron emission tomography, radiation detector, Monte Carlo simulation

## Abstract

Energetic electrons traveling in a dispersive medium can produce Cerenkov radiation. Cerenkov photons’ prompt emission, combined with their predominantly forward emission direction with respect to the parent electron, makes them extremely promising to improve radiation detector timing resolution. Triggering gamma detections based on Cerenkov photons to achieve superior timing resolution is challenging due to the low number of photons produced per interaction. Monte Carlo simulations are fundamental to understanding their behavior and optimizing their pathway to detection. Therefore, accurately modeling the electron propagation and Cerenkov photons emission is crucial for reliable simulation results. In this work, we investigated the physics characteristics of the primary electrons (velocity, energy) and those of all emitted Cerenkov photons (spatial and timing distributions) generated by 511 keV photoelectric interactions in a bismuth germanate crystal using simulations with Geant4/GATE. Geant4 uses a stepwise particle tracking approach, and users can limit the electron velocity change per step. Without limiting it (default Geant4 settings), an electron mean step length of ~250 μm was obtained, providing only macroscopic modeling of electron transport, with all Cerenkov photons emitted in the forward direction with respect to the incident gamma direction. Limiting the electron velocity change per step reduced the electron mean step length (~0.200 μm), leading to a microscopic approach to its transport which more accurately modeled the electron physical properties in BGO at 511 keV. The electron and Cerenkov photons rapidly lost directionality, affecting Cerenkov photons’ transport and, ultimately, their detection.

Results suggested that a deep understanding of low energy physics is crucial to perform accurate optical Monte Carlo simulations and ultimately use them in TOF PET detectors.

## 1 INTRODUCTION

Following a gamma interaction in a dispersive medium, energetic electrons are emitted such that their initial trajectories are mainly in the forward direction with respect to that of the incident gamma [1,2]. However, since the inelastic mean free path of energetic electrons in a dense material (e.g., 0.5 MeV and Z = 73) is in the order of a fraction of microns (~0.100–0.250 μm) [3], their path is extremely tortuous.

When traveling, these electrons asymmetrically polarize the medium forward and backward. Upon returning to their ground state, the atoms from the medium emit electromagnetic radiation that moves away spherically at the phase velocity of the medium, according to the Huygens principle [4]. If a charged electron moves through the dispersive medium of refractive index *n* with a velocity *v* greater than the speed of light in that medium (*c*/*n*), the emitted waves add up constructively, giving rise to coherent radiation known as Cerenkov radiation. Cerenkov radiation is emitted along the path of the energetic electron at a specific angle with respect to the electron direction, determined by the electron velocity, and inversely proportional to the medium refractive index [5]. Consequently, the first Cerenkov photons are emitted in a forward direction. However, due to the tortuous electron path in the medium, the emitted Cerenkov photons are expected to lose their directionality quickly.

Most materials used in gamma-ray detection have high refractive indexes (1.81 for lutetium-yttrium oxyorthosilicate (LYSO), ~2.15 for bismuth germanate (BGO), ~2.5 for thallium bromide (TlBr)), allowing high-energy electrons ejected as a result of both photoelectric and Compton interactions to reach the Cerenkov production threshold (*v* > *c*/*n*). Following a 511 keV interaction, these electrons can give rise to a few tens of Cerenkov photons [6]. The electron emission and propagation are extremely fast [7], and Cerenkov photons are emitted quasi-instantaneously, within a few ps [8]. The ultra-fast emission combined with the preferential forward direction of the first Cerenkov photons emitted makes them extremely promising to improve radiation detector timing resolution.

Using promptly emitted Cerenkov photons to achieve superior timing resolution in time-of-flight positron emission tomography (TOF PET) has been the core of several recent studies [9-16]. These studies underscored the challenges associated with time triggering based on the very few Cerenkov photons produced per photoelectric interaction and indicated the need for a more detailed understanding of the light transport in the crystal and collection by the photodetector. In this context, Monte Carlo simulation is an indispensable tool to access information not captured experimentally.

In previous works, we used advanced optical Monte Carlo simulation to study the transport and detection of Cerenkov photons in BGO and TlBr [17,18], which are both good candidates for triggering detectors on Cerenkov emission [13,19,20]. In agreement with experimental results, simulations showed that Cerenkov photons constitute most of the signal rising edge and are therefore critical to providing early triggering and improving timing resolution. In particular, we showed that the Cerenkov photon emission time is negligible compared to the transport time, making the Cerenkov detection time very sensitive to the emission direction and transport compared to the scintillation photon detection time [17].

**FIGURE 1 ∣:**
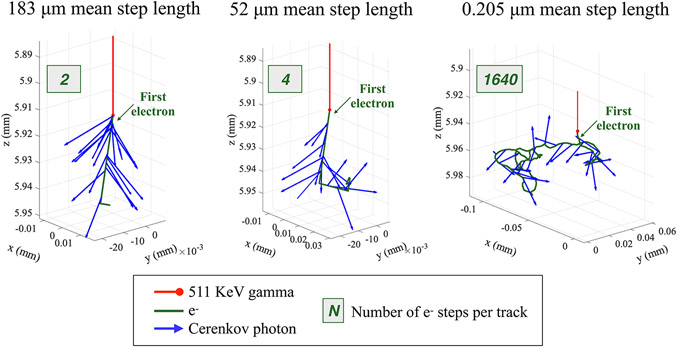
Examples of an electron track (green) within a BGO crystal generated by a 511 keV gamma (red) simulated in GATE/Geant4. Cerenkov photons (blue arrows) are produced along the electron track. **(A)** Using Geant4 default setting, the electron mean step length was of 183 μm, and they performed in average 2 steps per track. Most of the Cerenkov photons were emitted in the forward direction. **(B)** When imposing a 10% limitation on the electron velocity change per step, the electrons scattered 4 times in average and their mean step length was reduced to 52 μm. **(C)** By strongly limiting the electron velocity change per step to 0.04%, the mean step length was reduced to 0.205 μm and both the electrons and the Cerenkov photons lose their directionality. This figure shows the number of e^−^ steps per track of the specific tracks depicted.

Here, we investigate the propagation of energetic electrons and emission of Cerenkov photons in BGO using Geant4/GATE [21]. By default, in Geant4 simulations, the electron step length between interactions with the medium is randomly sampled from the electron range, itself computed from the physics cross-sections and the atom density in the medium. Figure 1A shows a qualitative example obtained using Geant4 default settings: a mean step length of 183 μm is obtained, with most of the Cerenkov photons emitted in the forward direction. However, the continuous energy loss and multiple scattering of an electron in a dense medium imposes a limit on its step length [3]. In Monte Carlo simulation with Geant4, the step length of an electron generated by a gamma interaction can be limited by imposing an upper bound on its velocity change per step. Figure 1(B-C) qualitatively shows examples of electron and Cerenkov photon trajectories when limiting the electron velocity change per step to 10 and 0.04% (representative values of velocity change in a step): the mean step length was reduced to 52 and 0.205 μm, respectively. Aside from contrasting these simulated values with the literature, there is no clear information on how to appropriately limit the velocity change per step.

While the primary electrons were always generated forward with respect to the gamma direction, the shorter the steps, the more interactions, and the less the electron directionality was maintained. Since the momentum of Cerenkov photons depends on that of their parent electrons, the electron step length is expected to directly affect their emission, transport in the crystal, and ultimately their detection.

Understanding and accurately modeling the emission and transport of electrons is thus a key point to perform accurate simulation of Cerenkov photons and ultimately use them to improve detector timing resolution. In this work, we investigated the emission of electrons and Cerenkov light in BGO using Monte Carlo simulations with Geant4/GATE. We thoroughly studied the primary electron and Cerenkov photon characteristics when limiting the electron step length. We qualitatively compared the simulated electron inelastic mean free path in BGO with its expected value from the literature [3].

## 2 MATERIALS AND METHODS

### 2.1 Propagation of Energetic Electrons and Emission of Cerenkov Photons in Geant4

**FIGURE 2 ∣:**
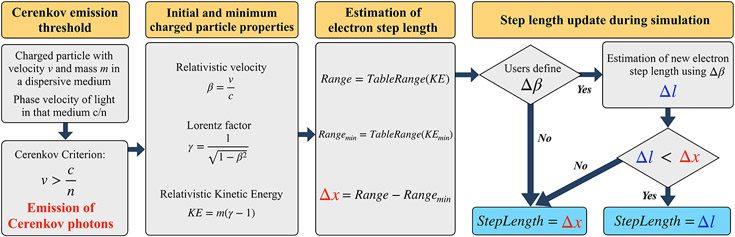
Schematic view of Geant4 steps to estimate the electrons’ step length when the emission of Cerenkov photons is allowed.

In GATE, the electron interactions and the generation of both scintillation and Cerenkov photons are based on the physics models implemented in Geant4 (Figure 2) [22]. More details can be found in the Geant4 physics manual [23].

Following a 511 keV interaction, high-energy primary electrons are emitted by both photoelectric and Compton interactions. Then these electrons lose their energy in the medium. The electron energy loss (*TableDeDx*) is tabulated using the physics process cross-sections extracted from a set of publicly distributed data libraries^1^. The energy loss tables are used to calculate the range table (*TableRange*) in multiple materials during the initialization phase of Geant4. These two tables are then used during the simulations. Some part of the electron energy loss due to inelastic collisions is continuous, and some part contributes to the production of secondary electrons. The production threshold is defined as the minimum energy above which these secondary electrons are explicitly produced and tracked.

When a recoil electron from a photoelectric or a Compton interaction traverses a dispersive medium with a velocity *v* exceeding the effective speed of light in that medium (*c*/*n*), the emission of Cerenkov photons is allowed (Figure 2, “*Cerenkov emission threshold*”).

#### 2.1.1 Electron Step Length Computation

Each electron is tracked step by step. One electron track is composed by multiple steps. The electron relativistic velocity *β*, the corresponding Lorentz factor *γ*, and its kinetic energy KE are computed at the beginning of each step and at the minimum energy (Figure 2, “*Initial and minimum charged particle properties*”). The minimum energy is set by the user-specific material optical properties (*Material.xml*). Using this information, each rectilinear step length (*Δx*) is calculated from the tabulated particle range (Figure 2, “*Estimation of electron step length*”).

By default, Geant4 uses *Δx* as the electron step length. However, users can limit the maximum % change of the charged particle velocity *β* per step^2^ (*Δβ*) (Figure 2, “*Step length update during simulation*”). If *β* represents the electron velocity at the beginning of an electron step, its velocity at the end of the step *β*_*f*_ is estimated using Equation 1.


(1) $$\beta_{f}=\beta \left(\;1-\Delta \beta \right)$$

Setting a limit to the electron velocity change per step *Δβ* limits the variation of the kinetic energy *ΔKE* by controling the Lorentz factor variation *Δγ* and consequently the electron step length *Δl* (Equations 2-4). A small *Δβ* value (e.g., 0.04%, 10%) leads to a small variation of Δ*γ* (Equation 3), a small variation of the kinetic energy Δ*KE* per step, and a small step length *Δl* (Equation 4; Figures 1B,C). In contrast, when using large *Δβ* values (e.g., 80%, 90%, or default Geant4 settings), the electron velocity can vary much more at each step, and, consequently, the electron travels in longer steps (Figure 1A).

(2) ΔKE=mΔγ

where:

(3) $$\Delta \gamma =\frac{1}{\sqrt{1-\beta^{2}}}-\frac{1}{\sqrt{1-\beta^{2}{\left(1-\Delta \beta \right)}^{2}}}$$

(4) $$\Delta l=m\frac{\Delta \gamma }{dE∕dx}$$

*m* is the electron mass (0.511 MeV), and *dE*/*dx* is the energy loss per step estimated from the electron total continuous energy loss tables. If this new step length *Δl* is smaller than the physical particle range *Δx* Geant4 stores *Δl* as the particle step length. If not, *Δx* is used as the step length instead (Figure 2, “*Step length update during simulation*”).

#### 2.1.2 Number of Emitted Cerenkov Photons

Once the charged particle step length is estimated, the number of emitted Cerenkov photons is calculated, and their physical characteristics are computed. The emission time and position of individual Cerenkov photons are calculated from quantities known at the beginning of each electron step.

The theoretical number of Cerenkov photons emitted by a single charged particle is calculated according to the theory of Cerenkov radiation of Frank and Tamm [5]. The number of photons *dN*/*mm* generated per unitary track length by an energetic charged particle with a relativistic velocity *β* in 1 mm of a material with an index of refraction *n* varying as a function of the Cerenkov energy *ε* is given by Equation 5:

(5) $$\frac{dN}{mm}=\frac{2\pi \alpha z^{2}}{hc}\int_{\varepsilon_{min}}^{\varepsilon_{max}}\left(1-\frac{1}{\beta^{2}n^{2}(\varepsilon )}\right)d\varepsilon$$

Here, *α* is the fine-structure constant (1/137), *z* is the charge of the particle (1 for electron), *h* is the Planck constant, and *ε* is the energy of the Cerenkov photon. Important to note that dN/mm depends on the Cerenkov energy spectrum [ε_*min*_ − ε_*max*_] defined by the user in the materials. Cerenkov photons emission is predominantly observed in the electromagnetic spectrum visible region [24]. Consequently, all the optical properties defined by the users should be restricted to the necessary optical energy range or the material physical Cerenkov energy range (cut-off) to avoid non-physical results, as this is not restricted by Geant4 [25].

The theoretical number of Cerenkov photons *dN*/*dx* emitted in a specific step length *dx* is calculated using Equation 6 and is invariant when dividing a step into smaller steps [26].


(6) $$\frac{dN}{dx}=\mathrm{Step}\mathrm{Length}*\frac{dN}{mm}$$

Since a variation of the electron velocity *Δβ* during a step affects its step length (Equation 4), it will also affect the number of Cerenkov photons generated in each step. The final number of Cerenkov photons emitted per step is a random variable following a discrete Poisson distribution of mean *dN*/*dx* in Geant4. The Cerenkov photons are produced evenly along the rectilinear track of the energetic electron. Their momentum direction depends on their relativistic velocity and the refractive index of the medium through the relation:

(7) $$\cos\;\theta =\frac{1}{\beta n(\varepsilon )}$$

Cerenkov photons, therefore, have a specific geometric signature since they are emitted along a cone with an angle *θ* with respect to the charged particle direction.

### 2.2 Electron and Cerenkov Photon Tracking Following 511 keV Gamma Interactions in BGO

#### 2.2.1 Simulation Set-Up

To investigate the emission, spatial distribution, and timing distribution of electrons and Cerenkov photons as a function of the electron velocity change per step, we performed optical Monte Carlo simulation in a BGO crystal with the toolkit GATE v9.0 (Geant4 10.06.03). BGO scintillation properties were defined as an isotropic emission following a single exponential decay with a time constant of 300 ns, a broad emission spectrum with a peak at 480 nm, and an index of refraction between 2.36 and 2.07, for energies between 320 and 800 nm. The absorption length was modeled in the same range [25]. The fast decay component of BGO (10% of the emission) and the rise time were not included. More details can be found in our previous work [17].

Simulations were conducted using a 511 keV monoenergetic source located at 10 mm from the center of a 3 × 3 × 20 mm^3^ BGO crystal, perpendicular to the 3 × 3 mm^2^ face. The photodetector was placed between the source emission and the crystal (front-arrangement). It was considered ideal, with a photon detection efficiency (PDE), which includes geometrical and quantum efficiency, of 1. A total of 1,000 gammas were emitted per simulation; only photoelectric interactions with the crystal were considered (photoelectric attenuation coefficient of ~0.056 cm^3^/g at 511 keV [27]. The optical photons produced by scintillation and Cerenkov emission from energetic electrons were simultaneously generated and tracked using the Livermore Model [28]. The transport of optical photons was conducted using the LUT Davis model [29-33] with polished surfaces LUTs wrapped in Teflon, except the face in contact with the photodetector, which was modeled coupled to optical grease (index of refraction 1.5) for all simulations.

We investigated the effect of the maximum electron velocity change per step *Δβ* with the following values: from 0.01 to 0.05% with 0.01% increments, 0.1, 0.5, 1, 5%, and from 10 to 90% in 10% increments. We also performed simulations using the default Geant4 settings, which do not limit the electron velocity change per step and compute the step length from the range table (see 2.1.1).

Two additional investigations followed the described simulations and studies. First, we carried out simulations in BGO using a 1,000 keV monoenergetic source. Second, we used TlBr as material and 511 keV gammas. The same material characteristics as in our previous works and a 440–800 nm range to define the optical properties were used [18,34].

#### 2.2.2 Simulation Output

In all simulations, the characteristics of all *emitted* electrons and optical photons were stored in text files. The electron kinetic energy at the beginning of each step, the step length, and the number of electron steps per track were saved. We included a tag to identify the nature of each optical photon emitted by the electrons using custom Geant4 source code modifications. This allowed the scintillation and Cerenkov photons that originated from the same electron to be discriminated. We stored the number of generated Cerenkov photons for each electron step. The momentum of the primary electron and that of the *i*^th^ emitted Cerenkov photon (*i* varying from 1 to 11) were also saved.

The spatial and timing information of the *detected* optical photons was saved in ROOT files [35].

#### 2.2.3 Analysis

The text and root output files were analyzed with MATLAB^®^ to reconstruct the complete history of each electron, identifying the parent gamma event and separating the scintillation and Cerenkov photons emitted at each step.

First, we investigated the effect of limiting the electron velocity change per step *Δβ* on the electron kinetic energy at the beginning of each step, step length, and number of steps in each track.

Using the step lengths of each electron track, we calculated the electron mean step length and mean track length, as the average of the mean step length per track over all tracks and the average of the summed step length per track over all tracks, respectively. We studied the obtained mean step length and track length as a function of the electron velocity change per step *Δβ*. We qualitatively compared the simulated mean step lengths with the mean free path of electrons in BGO.

Second, we studied the mean number of Cerenkov photons emitted per 511 keV interaction as a function of the electron mean step length, and consequently to the user-defined *Δβ*. Considering one 511 keV event, the number of Cerenkov emitted resulted from summing the number of Cerenkov photons per electron step over all steps. The mean number of emitted Cerenkov photons per 511 keV was extracted from the distribution for all events, using a Gaussian fit.

Then, we showed the relationship between the electron mean step length and the primary electron and the *i*th emitted Cerenkov photon directionality for all photoelectric interactions. Their momentum at the emission was projected on the x-z plane (Figure 1 as reference), and the correspondent angle with respect to the gamma emission was extracted.

Lastly, we investigated how the spatial and timing distributions of the detected Cerenkov photons within the 20 mm BGO are affected by different mean step lengths of the electron.

## 3 RESULTS

### 3.1 Electron Properties

#### 3.1.1 Electron Track and Kinetic Energy

**FIGURE 3 ∣:**
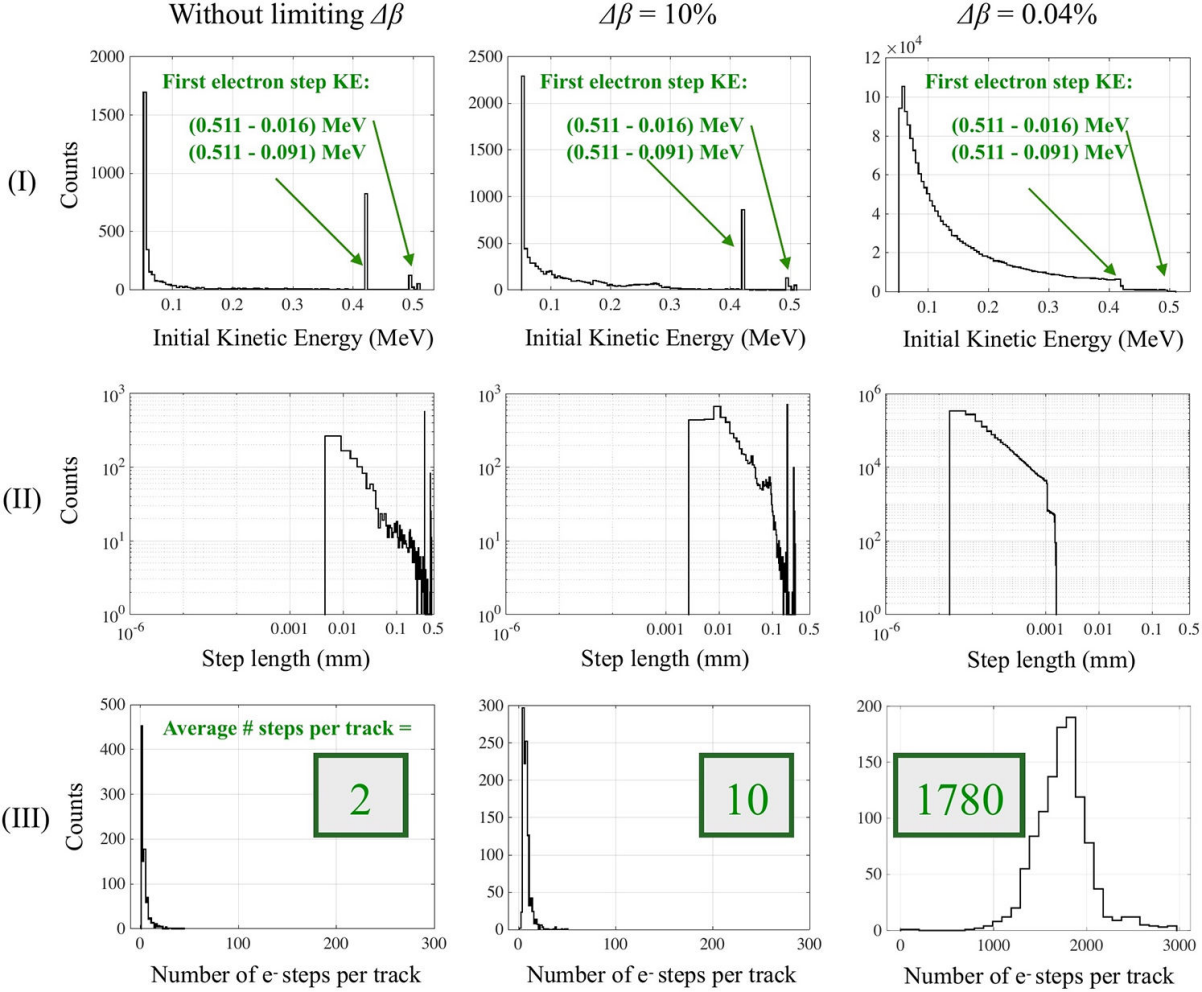
(I) Electron initial kinetic energy at the beginning of each electron step, where the kinetic energy at the beginning of the very first electron step is highlighted in green. (II) Electron step length (in log-log scale) and (III) number of step lengths per track. Results are shown for all tracks and limiting the maximum change of the electron velocity during a step (*Δβ*): **(A)** no value of *Δβ* (default Geant4 setting) is used, **(B)** a limit of 10% or **(C)** 0.04% is set on the electron velocity change per step. The *y*-axes scales are different in all figures.

We studied the electron kinetic energy at the beginning of each step, the step length (all steps in all tracks) and the number of electron steps per track for three representative values of *Δβ* (Figure 3).

Without limiting *Δβ* (default Geant4 setting), each primary emitted electron had a kinetic energy of 0.420 MeV at the beginning of its track (Figures 3A,I). This corresponds to the kinetic energy of a recoil electron generated by the photoelectric interaction of a 0.511 MeV gamma with the 0.091 MeV K-edge of bismuth in BGO [27]. A minority of electrons interacted with the second and third energy orbits of bismuth (L-M shells, 0.001–0.016 MeV) and had an initial kinetic energy of ~0.5 MeV. The majority of these 0.42 and 0.5 MeV electrons traveled ~0.348 mm and 0.435–0.455 mm during this first step of their track (Figures 3A,II). On average, they performed only two steps per track (Figures 3A,III). They lost most of their energy during the first “long” step, and consequently, the following steps are only a few microns long. This is evidenced by the low peaks of the kinetic energy spectrum and step length distribution (Figure 3A (I-II)).

The same results are obtained with *Δβ* equal to or greater than 30% (not shown). This indicates that the corresponding step length *Δl* estimated from Equation 4 is generally larger than the physical step length *Δx* estimated from the electron range table, as discussed in Section 2.1.1 and summarized in Figure 2. Geant4 therefore always equals the electron step length to *Δx* for *Δβ* greater than 30%. Results obtained without limiting *Δβ* and with *Δβ* greater than 30% are therefore considered together in the rest of this paper.

When limiting *Δβ* to 10% and further to 0.04%, the kinetic energy at the beginning of each electron track was always given by the photoelectric interaction of the 511 keV gammas (Figure 3(B-C) (I); note the different *y*-axis scales in Figure 3I). However, *Δβ* set a limit on how much the electron velocity could vary per step. This led the electrons to scatter a greater number of times, traveling multiple shorter steps, and losing less energy at each step (Figure 3(B-C)).

#### 3.1.2 Electron Mean Step Length per Track and Mean Track Length

Using all step lengths of each electron track, we calculated the electron mean step length and mean track length.

**FIGURE 4 ∣:**
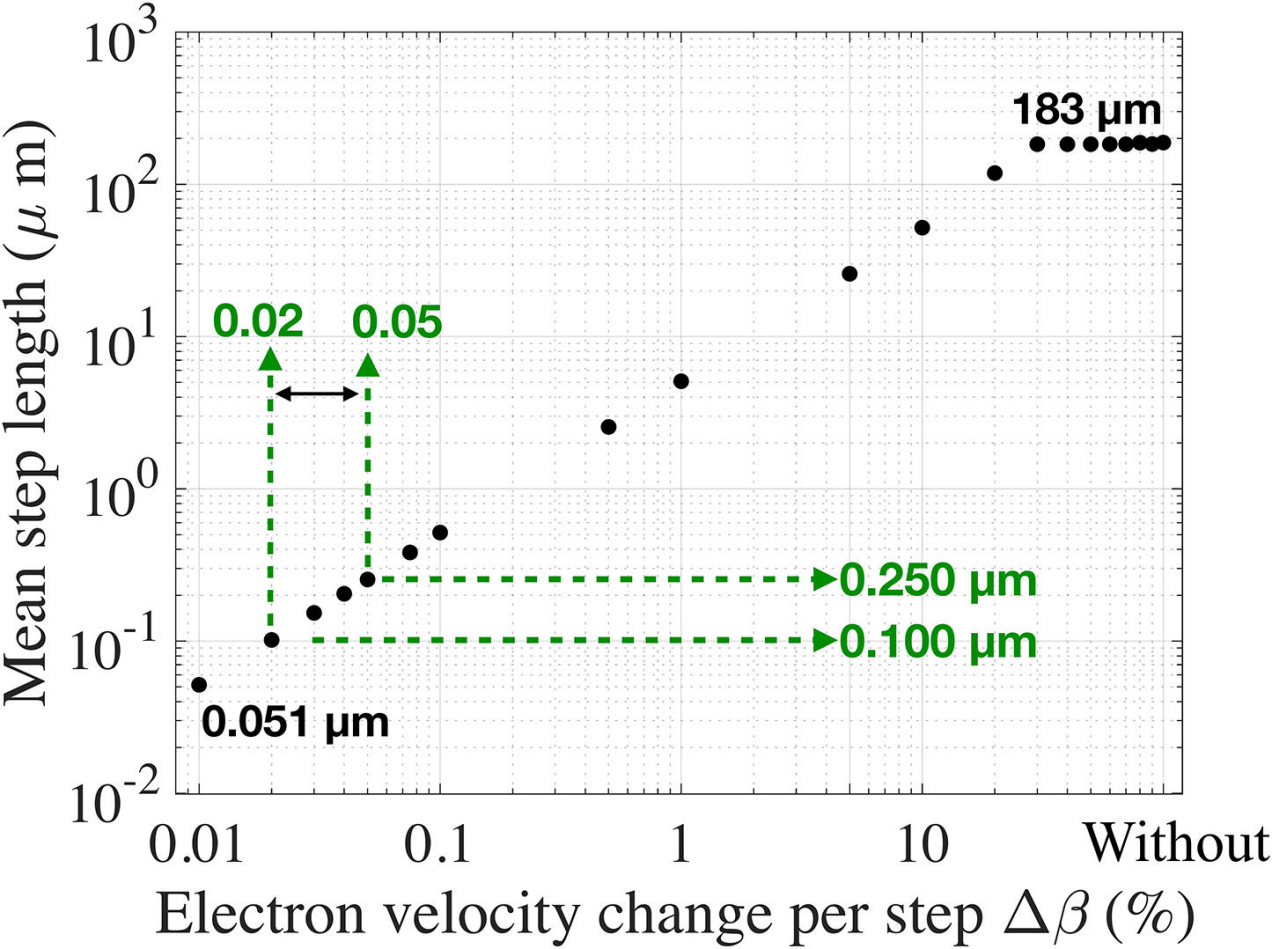
Electron mean step length as a function of the electron velocity variation *Δβ*, in log-log scale. Shown in green is the *Δβ* range and corresponding mean step length that best approximate the electron mean free path in BGO.

There is a clear linear relationship between *Δβ* and the mean step length per track (Figure 4, in log-log scale). For *Δβ* between 0.01 and 30%, the mean step length increased from 0.051 to 183 μm and plateaued for larger *Δβ* values.

We compared these values with the electron mean free path calculated with the relativistic equation of electron interactions with bulk materials [3,36]. The mean free path of a ~400 keV electron in a solid bulk material with Z equal to 73 mainly composed of bismuth or germanium (akin to BGO), was estimated to be ~0.170 μm. The calculation was validated with measurement up to 200 keV. From 511 keV to 1 MeV, the authors estimated the reported values to be within 0.2% of the correct values.

Based on this assumption, a reasonable electron mean free path in BGO at 511 keV should fall between 0.100 and 0.250 μm (Figure 4), which is obtained with an electron velocity change per step *Δβ* between 0.02 and 0.05%. The electron velocity change per step has no effect on the electron mean track length (Supplementary Figure S1).

### 3.2 Number of Emitted Cerenkov Photons as a Function of the Electron Mean Step Length

**FIGURE 5 ∣:**
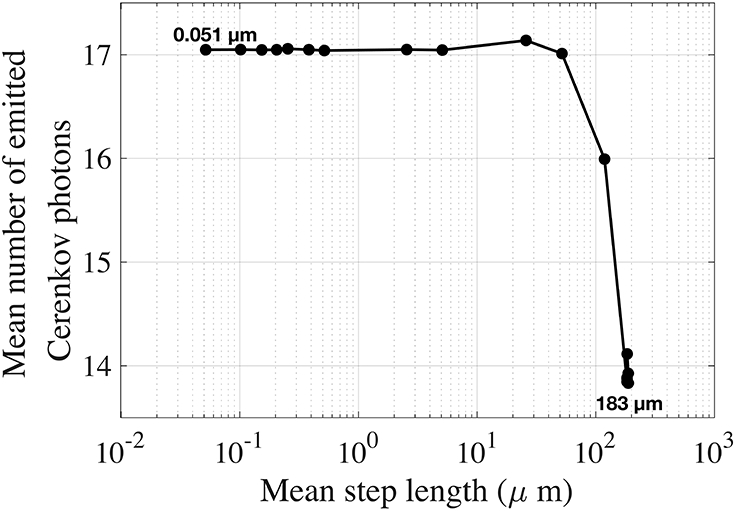
Mean number of emitted Cerenkov photons per 511 keV photoelectric interaction in BGO as a function of the electron mean step length. The electron mean step length is varying in the simulations since different limitations on the electron velocity change per step are imposed.

We studied the mean number of emitted Cerenkov photon per 511 keV interaction as a function of the electron mean step length (Figure 5, log scale), using values calculated in the previous section (Figure 4).

For an electron mean step length between 0.051 and 52 μm, the mean number of emitted photons per photoelectric interaction was 17. Between 52 and 183 μm, the mean decreased to 14 and remained constant for longer mean step lengths. For a given interaction, the number of emitted Cerenkov photons is not expected to vary with the electron mean step length. The observed decrease for large mean step length could be due to estimating the number of emitted Cerenkov photons using the Frank-Tamm formula assuming that the electrons travel long step with a constant velocity, which is not physically accurate.

Considering that the electron mean free path range in BGO at 511 keV should be between 0.100 and 0.250 μm as discussed in the previous section, the mean number of emitted Cerenkov is expected to be 17. This value is consistent with experimental results, which report an average value of 17 ± 3 [37].

Since the Cerenkov photon momentum depends on that of its parent electron, the electron step length was expected to directly affect the Cerenkov directionality upon emission, as shown in next section.

### 3.3 Electron and Cerenkov Photon Directionality

#### 3.3.1 Primary Electron, and First Emitted Cerenkov Photons’ Momenta at Emission

**FIGURE 6 ∣:**
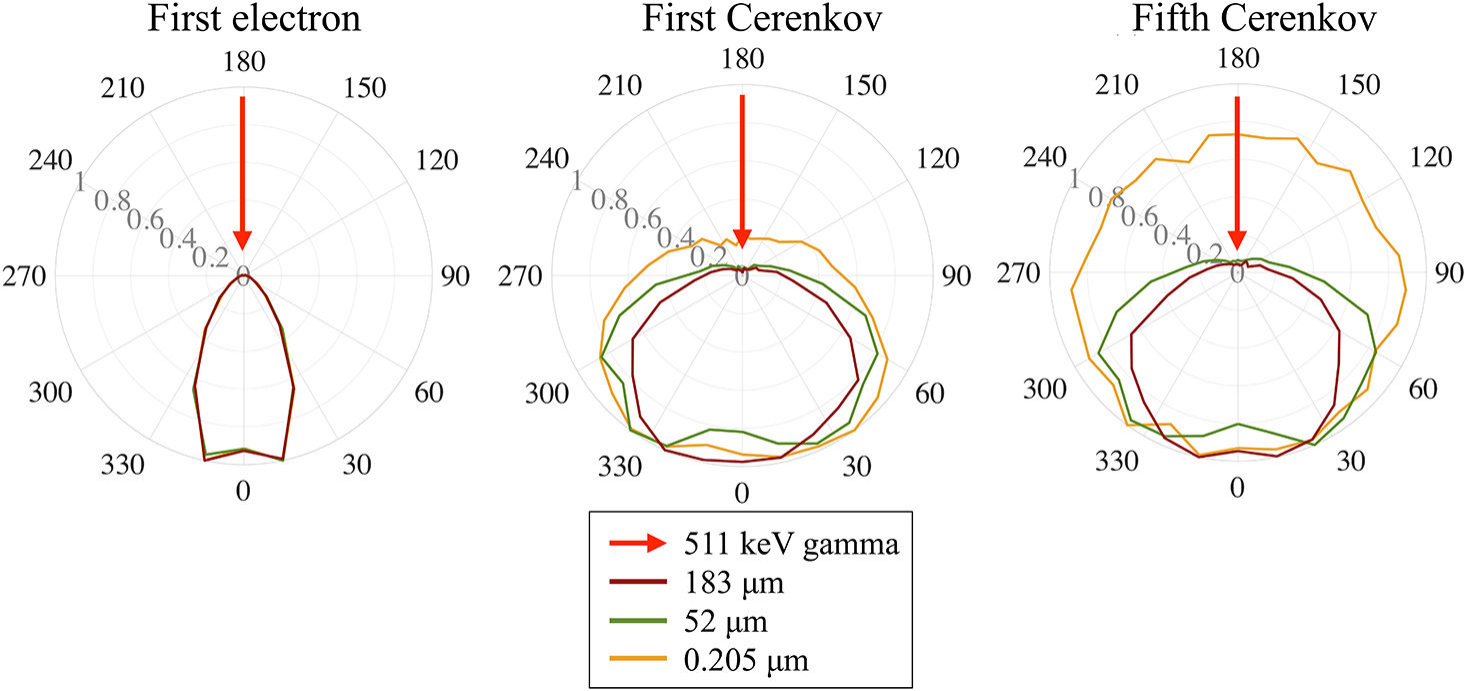
Polar plots of the normalized distribution of the **(A)** first electron **(B)** first and **(C)** fifth emitted Cerenkov photon momentum with respect to the incident gamma direction, projected on the x-z plane according to Figure 1, for three representative values of the electron mean step length. The 0–1 scale represents the magnitude of the polar plot.

Figure 6 shows the angular distribution of the primary electron, the first and fifth emitted Cerenkov photons at their emission, for each photoelectric interaction and for three mean step lengths values, which correspond to the three representative values of *Δβ* showed in Figure 3. Each angular distribution was normalized to its maximum to highlight preferred directions of emission. The directionality is also illustrated in Figure 1.

Figure 6A shows the angular distribution of the primary electron momentum at emission. As expected, following a gamma interaction, the energetic electrons are emitted in the forward direction, independently of the length of their steps.

Since the emitted Cerenkov photons’ momentum varies with respect to that of their parent electron (Eq. 7), the momentum distribution of the first emitted Cerenkov photon favors forward directions (Figure 6B).

Shorter electron steps make the track more tortuous and weaken the electron directionality with respect to the incident gamma direction. This effect is directly visible from the momentum distribution of the fifth Cerenkov photon emitted (Figure 6C). When the mean electron step was 183 μm, the electrons traveled only a few steps (Figure 3) and the fifth Cerenkov photon was emitted within the first electron step, thus maintaining the forward direction information. When the mean electron step decreased to 52 μm, the first electron step was still long enough to include the fifth emitted Cerenkov photon and maintain the forward direction. In contrast, when the electron mean step length was in the order of 0.200 μm, the electron rapidly lost directionality, causing the Cerenkov photons to lose their directionality.

#### 3.3.2 I^th^ Emitted Cerenkov Photon Distributions at 180°

**FIGURE 7 ∣:**
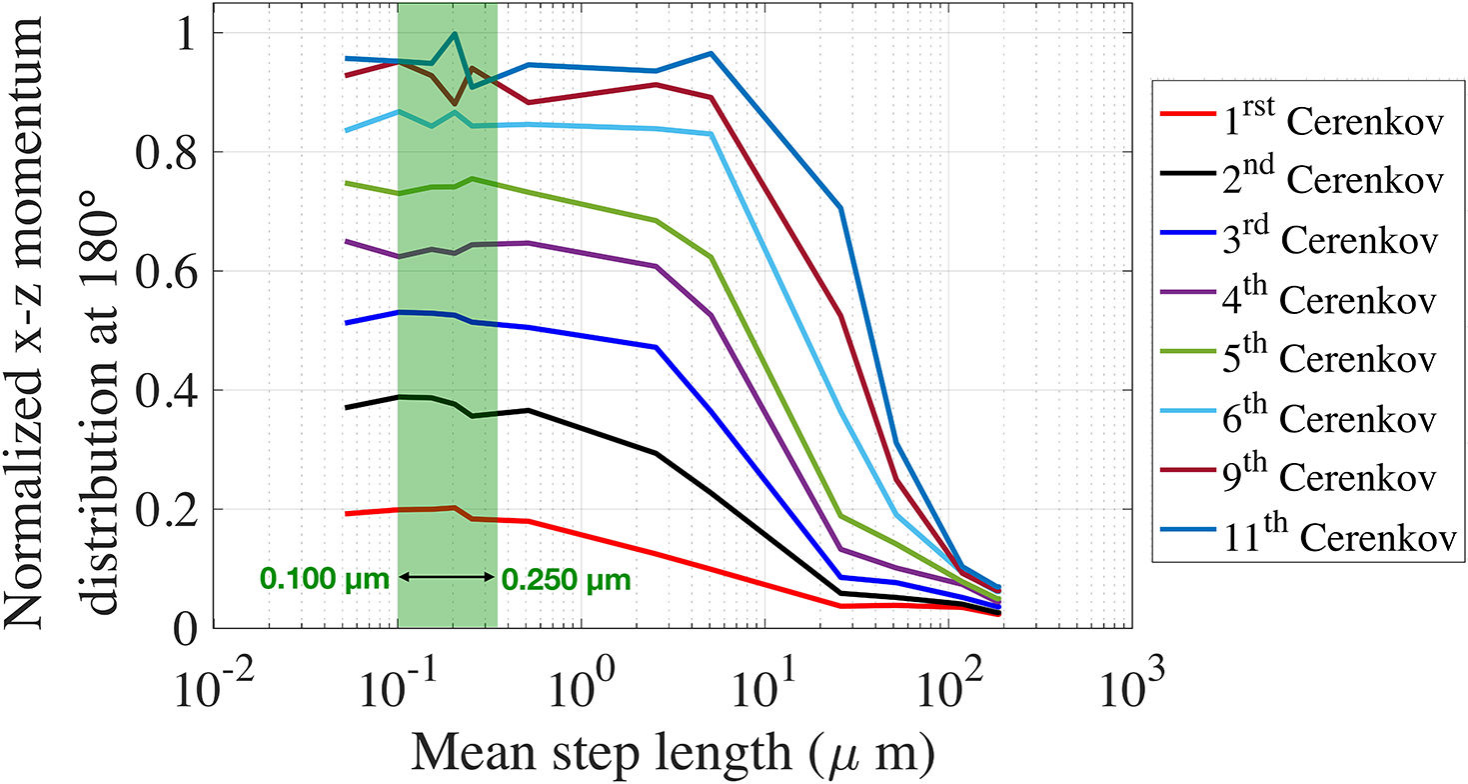
Distribution of the *i*^th^ emitted Cerenkov photons momentum at 180° compared to the gamma direction, projected on the x-z plane, as a function of the electron mean step length.

We investigated subsequently emitted Cerenkov photons up to the 11^th^ photon. Figure 7 (*x*-axis in logarithmic scale) shows the normalized distribution of the x-z photon momentum at 180° with respect to the gamma emission (backward) for different electron mean step lengths.

For a given electron mean step length, the fraction of events with a x-z momentum component at 180° was larger for Cerenkov photons emitted later, meaning that the forward directionality decreased (also illustrated in Figure 1). This effect was more significant for small electron mean step lengths (< 10 μm). It became weaker for larger mean step lengths (e.g., >100 μm, Figure 7) since the first eleven Cerenkov photons were all emitted forward during the first electron step.

The x-z momentum distribution in the 0.100–0.250 μm range (electron mean free path in BGO at 511 keV) was almost constant for all Cerenkov photons (green region highlighted in Figure 7). Therefore, all electron mean step length values in this range led to a similar Cerenkov emission distribution during the simulation. Note that the 0.100–0.250 μm polar momentum distributions for the first and fifth Cerenkov photons correspond to the yellow distributions in Figure 6.

These results directly affect the Cerenkov photon transport within the material, as shown in the next subsection.

### 3.4 Effect on Cerenkov Photon Transport

**FIGURE 8 ∣:**
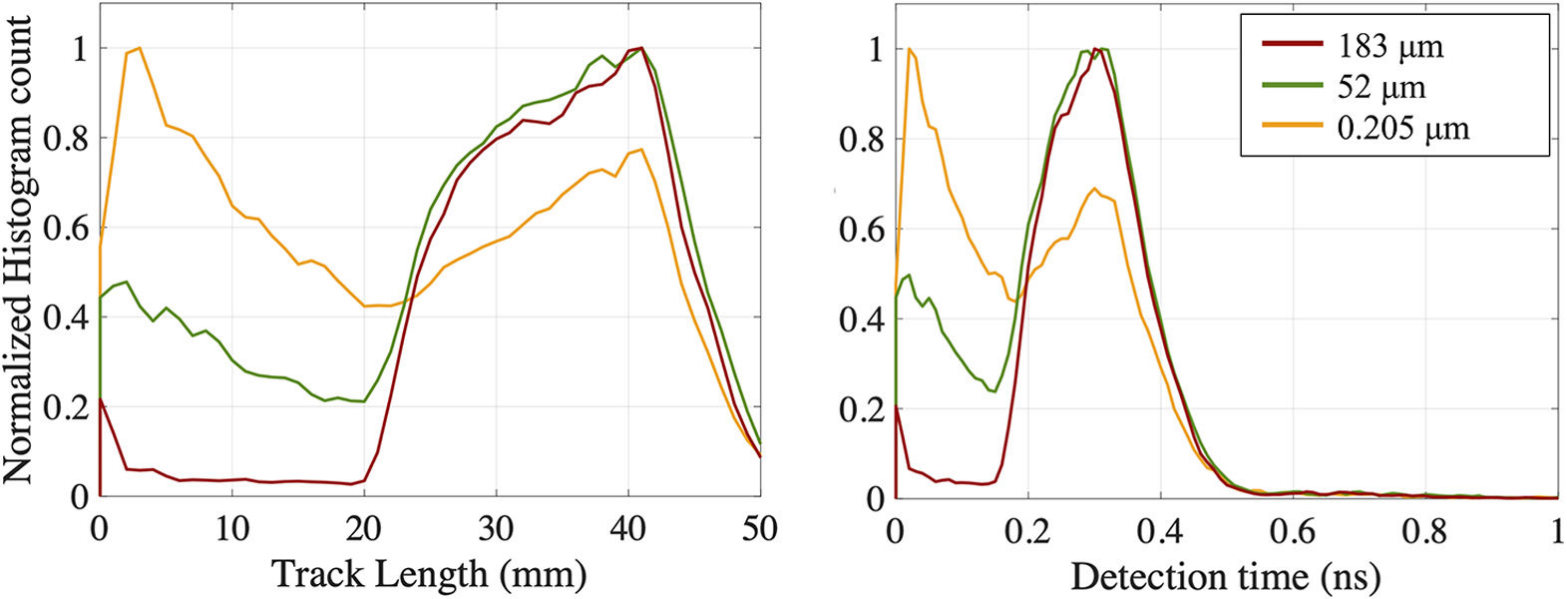
Cerenkov photons **(A)** track length and **(B)** detection time distributions, for three electron mean step length values. Simulations were performed in a 20 mm thick BGO crystal, with the photodetector placed between the 511 keV gamma source and the crystal.

We studied the effect of simulating different electron mean step length on the detected Cerenkov photon track length and detection time in a 20 mm thick BGO wrapped in Teflon, with the photodetector placed between the 511 keV gamma source and the crystal. Results are shown in Figure 8 for three mean step length values.

With large mean step length (~183 μm, dark red line in Figure 8A), the majority of the Cerenkov photon were emitted forward (Figure 7) and needed at least one reflection on the crystal back face to reach the photodetector. Consequently, they traveled more than 20 mm within the crystal before reaching the photodetector and being detected.

When decreasing the mean step length (~52 μm, green line in Figure 8A), some electrons originally emitted forward lost their directionality through scattering in the crystal. The Cerenkov photons emitted forward at each electron step subsequently lost the directionality as well (Figure 1B and Figure 7). Hence, a non-negligible number of the photons emitted close to the photodetector face reached it with shorter paths traveling backward, as shown by an increasing number of events traveling only 1–10 mm.

The effect is stronger with small electron mean step length values (yellow line in Figure 8A) due to the increased number of backwards emitted Cerenkov photons (Figure 7).

The different Cerenkov photons distributions within the crystal were directly impacted by the electron mean step length (Figure 8B). A longer photon track length resulted in a later detection time. The photons traveled ~0.3 ns before being detected. The photon track lengths shortened when decreasing the mean electron step length, and so did the detection time (Figure 8B), with an average of less than 0.1 ns

### 3.5 Effect of Gamma Energy and Material

#### 3.5.1 Simulations in BGO at 1,000 keV

When using 1,000 keV gamma interaction, high energetic recoil electrons (~910 keV) are emitted by the photoelectric interaction with the K-edge of bismuth in BGO. By increasing the gamma energy, the electrons kinetic energy increased, and so did their relativistic velocity *β*, leading to longer step lengths.

**FIGURE 9 ∣:**
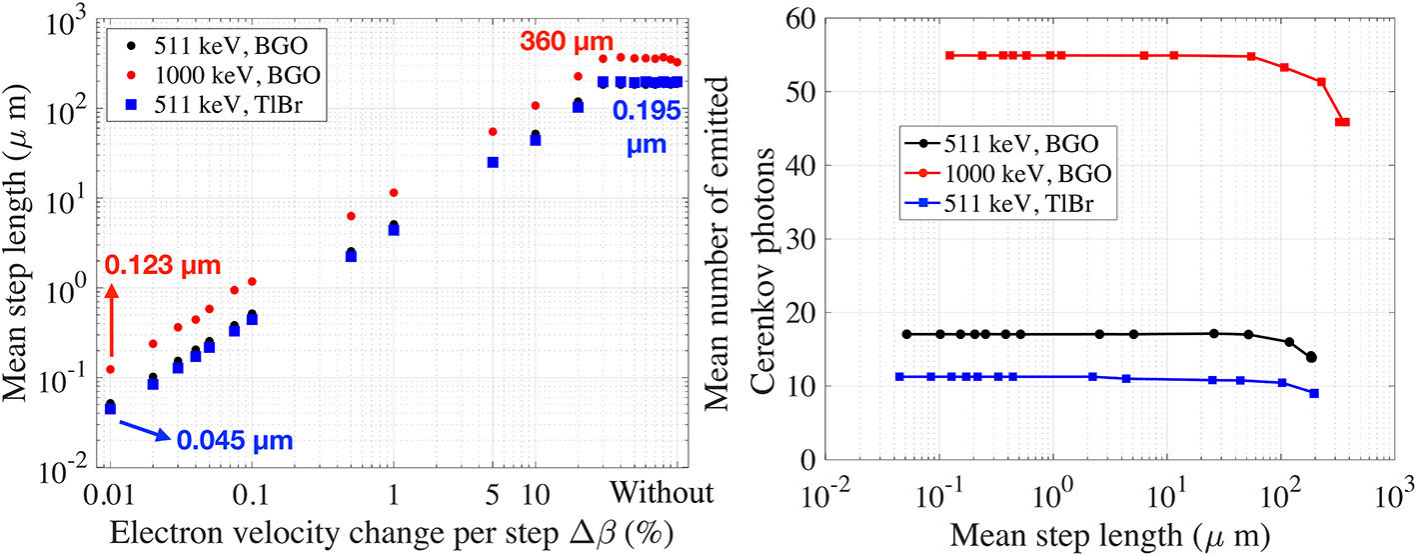
**(A)** Mean step length as a function of the electron velocity change per step, in logarithmic scale. **(B)** Mean number of emitted Cerenkov photons for photoelectric interaction as a function of the electron mean step length. Results are shown for BGO in its transmission range (320–800 nm), using 511 and 1,000 keV. TlBr was simulated within its transmission range at 511 keV (440–800 nm).

These energetic electrons traveled in average 360 μm when the electron velocity change per step *Δβ* was not limited (dots in Figure 9A), ~177 μm more than their mean path at 511 keV (black dots in Figure 9A, already shown in Figure 4). Between 0.01 and 30%, the mean step length increased from a minimum of 0.123 μm to a maximum of 360 μm, respectively. Considering previous electron mean free path calculations [3,36], at ~1,000 keV electrons travel around 200–300 μm in a solid bulk material composed by bismuth or germanium. Therefore, accurate results could be obtained using a *Δβ* of 0.02% or 0.03%, which corresponds to ~55 emitted Cerenkov photons (Figure 9B).

#### 3.5.2 Simulations in TlBr at 511 keV

In TlBr, the mean step length increased from 0.045 to 195 μm between 0.01 and 30% (blue dots in Figure 9A). The mean step length trend and values obtained with TlBr were similar to BGO (black dots in Figure 4 and Figure figure9A). This is probably due to their similar bulk characteristics (density and effective atomic number Z_eff_) [12]. For this reason, the *Δβ* values that best approximate the path of the electrons in TlBr at 511 keV are expected to be in the 0.02–0.05%, as with BGO at 511 keV, with electrons traveling 0.100–0.250 μm.

TlBr produced 11 Cerenkov photons per 511 keV interaction when the electron mean step length was smaller than 100 μm. As expected, the effect of *Δβ* on the number of emitted Cerenkov photons is material-specific (different optical properties) and should be investigated for each case by users.

## 4 DISCUSSION AND CONCLUSION

Our work aimed at studying the production of electrons and Cerenkov photons when gamma photons interact in high-density, highly refractive materials such as BGO and TlBr using Monte Carlo simulation with GATE/Geant4. We thoroughly investigated the effect of constraining the electron maximum relativistic velocity change per step on the electron mean step length and directionality and how it consequently affected the Cerenkov photons emission and detection.

We showed that reducing the electron velocity change per step yielded electron mean step length as small as 0.050 μm. Using higher to no limitation to the electron velocity change yielded longer electron step lengths, with electrons traveling 180 μm on average. We compared the obtained electron mean step length with the electron mean free path found in literature (~0.100–0.250 μm) [3,36]. We found that this range was obtained when limiting the electron velocity change per step to 0.02–0.05%.

These electrons emitted ~17 Cerenkov photons per 511 keV photoelectric interaction. These numbers were obtained when defining the BGO optical properties in the 320–800 nm energy range. Published experimental and simulated values of emitted Cerenkov photons report an average of 17 ± 3 at 511 keV in BGO [37]. Thus, our simulated values in this range are in good agreement with other reported results. The variation of the number of emitted Cerenkov photons with the velocity change raises questions on how Geant4 estimates the Cerenkov production (number and direction of the emitted photons). Further investigations to study and potentially improve the accuracy of the Cerenkov emission model are conducted and will be reported in a separate article focusing solely on Cerenkov photons.

Finally, we showed the effect of limiting the electron velocity change per step on the first electron and Cerenkov photons emission distributions. The emitted Cerenkov photons angular distributions at emission were highly sensitive to the electron mean step length and to their order of emissions. The differences in the momentum angular distribution at emission led to important differences in the Cerenkov photons transport and detection time in the crystal. We studied Cerenkov photons spatial and timing distributions using a 20 mm-thick BGO crystal with a photodetector in a front arrangement. This configuration was chosen to amplify the effect to better study it.

The directionality results are particularly important when using Monte Carlo simulations to optimize detectors timing resolution using Cerenkov photons, since the first Cerenkov detected is not always the first emitted and thus an accurate simulation of all of them is required.

We showed that the directionality distribution of each emitted Cerenkov photons was constant in the ~0.100–0.250 μm electron mean step length range, suggesting that each value in this range could lead to similar spatial and timing distribution.

We concluded that electron velocity change per step between 0.02 and 0.05% best approximated the electron physical properties in BGO at 511 keV. The range changes with the gamma energy and the material and no general absolute values and thresholds can be concluded from this study.

The most accurate electron path should be chosen by users for their specific application. Using a macroscopic model of the electron transport, with the step length estimated using the electron range, leads to less detailed modeling of Cerenkov photons emission and distribution in the material. One advantage of using a larger electron step length is the computational time up to 16 times faster (a few minutes compared to a few hours) and output files dimension up to 140 smaller (a few tens of MB compared to a few GB for the ROOT output). With a smaller electron step length, simulations are performed with a microscopic approach to the electron transport in the material, which leads to more accurate optical simulations of Cerenkov emission and transport and should be preferred when one wants to study the directionality and the timing distribution of these optical photons.

Although scintillation photons were simulated, we focused our attention on Cerenkov photons. The analysis of the differences between Cerenkov and scintillation photons distributions was shown in our previous work [17]. Due to the large number of scintillation photons emitted and their isotropic emission, the electron step length net effect on scintillation photons was negligible in this work and was not shown.

Geant4 has been originally developed to perform Monte Carlo simulations in high energy and nuclear physics. In these energy ranges from a few keV up to TeV, limiting the emission of optical photons by massive, charged particles is helpful in limiting the computational burden inherent to the stepwise particle tracking. In contrast, Geant4/GATE simulations at lower energies for medical applications using gamma emitters are much more sensitive to the accuracy of the electron step length. This is particularly critical when studying Cerenkov photons. Future studies will include experimental studies to investigate Cerenkov photons directionality in commonly used materials such as BGO, TlBr, and thallium chloride (TlCl).

Beyond the Cerenkov emission in BGO, several other cutting-edge studies for fast timing can be affected by the changes in the electron physics introduced by an accurate electron modeling. The use of meta-materials [38,39], which are extremely sensitive to the path of the first photoelectron, or Cerenkov charge induction detectors [18,40,40,41,41], sensitive to the cloud of electrons generated by the first photoelectron, are two examples.

The results shown here demonstrate that a deep understanding of the physics in the simulations is required to perform accurate optical simulations. This work aims to inform the Geant4/GATE user community of these effects and challenges in parameterizing optical simulations in radiation detectors.

## Supplementary Material

supplementary figures

## Data Availability

The raw data supporting the conclusion of this article will be made available by the authors, without undue reservation.

## Footnotes

1 Evaluated data sets are produced through the process of critical comparison, selection, renormalization and averaging of the available experimental data, normally complemented by model calculations [23]

2 It can be done using the *SetMaxBetaChangePerStep* command when generating Cerenkov photons. More details on how to set it can be found in [42].
