# Supplementary material for: The Accuracy of Cerenkov Photons Simulation in Geant4/Gate Depends on the Parameterization of Primary Electron Propagation
Source: Front Phys. Author manuscript; Available in PMC 2023 May 22. (PMC10201934; doi:10.3389/fphy.2022.891602)
Supplement: supplementary figures [file NIHMS1848555-supplement-supplementary_figures.docx]

**Supplementary Figure 1.** Electron mean track length as a function of the electron velocity variation *Δβ* , in log-log scale.
